# Expression and Regulation of Prostate Apoptosis Response-4 (Par-4) in Human Glioma Stem Cells in Drug-Induced Apoptosis

**DOI:** 10.1371/journal.pone.0088505

**Published:** 2014-02-11

**Authors:** Jayashree C. Jagtap, Parveen Dawood, Reecha D. Shah, Goparaju Chandrika, Kumar Natesh, Anjali Shiras, Amba S. Hegde, Deepak Ranade, Padma Shastry

**Affiliations:** 1 National Centre for Cell Science (NCCS), Pune, India; 2 Department of Neurosurgery, D. Y. Patil Medical College, Pune, India; Complutense University, Spain

## Abstract

Gliomas are the most common and aggressive of brain tumors in adults. Cancer stem cells (CSC) contribute to chemoresistance in many solid tumors including gliomas. The function of prostate apoptosis response-4 (Par-4) as a pro-apoptotic protein is well documented in many cancers; however, its role in CSC remains obscure. In this study, we aimed to explore the role of Par-4 in drug-induced cytotoxicity using human glioma stem cell line - HNGC-2 and primary culture (G1) derived from high grade glioma. We show that among the panel of drugs- lomustine, carmustine, UCN-01, oxaliplatin, temozolomide and tamoxifen (TAM) screened, only TAM induced cell death and up-regulated Par-4 levels significantly. TAM-induced apoptosis was confirmed by PARP cleavage, Annexin V and propidium iodide staining and caspase-3 activity. Knock down of Par-4 by siRNA inhibited cell death by TAM, suggesting the role of Par-4 in induction of apoptosis. We also demonstrate that the mechanism involves break down of mitochondrial membrane potential, down regulation of Bcl-2 and reduced activation of Akt and ERK 42/44. Secretory Par-4 and GRP-78 were significantly expressed in HNGC-2 cells on exposure to TAM and specific antibodies to these molecules inhibited cell death suggesting that extrinsic Par-4 is important in TAM-induced apoptosis. Interestingly, TAM decreased the expression of neural stem cell markers - Nestin, Bmi1, Vimentin, Sox2, and Musashi in HNGC-2 cell line and G1 cells implicating its potential as a stemness inhibiting drug. Based on these data and our findings that enhanced levels of Par-4 sensitize the resistant glioma stem cells to drug-induced apoptosis, we propose that Par-4 may be explored for evaluating anti-tumor agents in CSC.

## Introduction

High grade gliomas (HGG) or malignant gliomas are the most common of brain tumors in adults. Despite marked improvement in multimodality treatment, the overall prognosis of patients with HGG remains restrained corresponding to median survival period ranging between 9–12 months [1], [2]. Understanding and unraveling the biological basis of tumor formation and progression in gliomas is important for devising improved therapeutic strategies. Recent reports have shed light on a subpopulation of cells termed ‘cancer stem cells' (CSC) within solid tumors that compel tumor formation and growth [3]–[5]. Though many studies demonstrated that CSC are highly resistant to conventional chemotherapy and radiation therapy [6], [7], a recent review suggested that CSC are neither resistant nor sensitive to chemotherapy *perse* [8]. Much of the lacuna in understanding the role of CSC in conferring chemoresistance to gliomas is attributed to lack of appropriate experimental models. Glioblastoma multiforme (GBM) is regarded as a suitable model for the investigation of cancer stem cells [8]. We have recently reported a model system comprising of a long-term *in vitro* culture of human neuroglial culture (HNGC)-1 and an established cell line, HNGC-2, derived from the same human adult glioma tissue [9]. We have earlier reported detailed characterization of this cell line encompassing the essential features of cancer stem cells, which include the ability of self-renewal, the capacity to form CD133-positive neurospheres and develop intracranial tumors. Thus, HNGC-2 cell line serves as an ideal tool for studying glioma stem cells [10].

The prostate apoptosis response-4 (Par-4) is a tumor suppressor protein of approximately 38 kDa, encoded by PAWR gene (PKC apoptosis WT1 regulator) [11]. While Par-4 is expressed in normal and tumor cells [12], the significance of Par-4 in cancer cells is accredited to its proapoptotic function [11], [13]. Par-4 is silenced or downregulated either transcriptionally or post-transcriptionally in various types of cancers including gliomas [14]–[16]. Par-4 is downregulated during tumor recurrence in breast cancer and the downregulation is necessary and sufficient to promote recurrence [17]. Endogenous Par-4 is essential for sensitization of cells to diverse apoptotic stimuli, while the expression of Par-4 induced ectopically or triggered by anticancer drugs can selectively cause apoptosis in cancer cells [18]–[20]. In addition to its role as an intracellular proapoptotic protein, other studies have demonstrated that secretory or extracellular Par-4 also induces apoptosis in cancer cells and the mechanism involves binding of Par-4 to GRP78 [21].

Tamoxifen, a potent estrogen receptor (ER) antagonist derived from non-steroid triphenylethylene has been extensively used to treat ER-positive breast cancer. Recent studies suggest that high doses of tamoxifen can be beneficial in the treatment of gliomas [22], [23]. This effect is shown to be mediated by inhibition of protein kinase C (PKC) activity which is critical for proliferative signal transduction in gliomas [24]. Though TAM is being evaluated in clinical trials for treatment of patients with malignant gliomas [25], the effectiveness of tamoxifen on cancer stem cells or glioma stem cells has not been addressed.

In this study, we examined the sensitivity of glioma derived stem cell line - HNGC-2 and primary culture derived from glioma tumor samples that express neural stem cell markers (G1) to a panel of drugs including- lomustine, carmustine, UCN-01, oxaliplatin, temozolomide, tamoxifen (TAM) and the association of Par-4 with drug-induced apoptosis. We show that among the drugs tested only TAM induced cell death and upregulated Par-4 expression. Knock down of Par-4 protected the cells from TAM-induced apoptosis. Our data also demonstrate the involvement of secretory Par-4 and regulation of GRP78 in TAM-induced apoptosis.

## Materials and Methods

### Ethics Statement

The study was approved by the Ethics Committee of NCCS (Pune, India). Written consent was obtained from patients for use of tumor samples for research purposes.

### Reagents and Antibodies

Lomustine, carmustine, oxaliplatin, UCN-01, temozolomide, tamoxifen and GAPDH antibody were purchased from Sigma (USA). Antibody to GRP78 was from Abcam and Santa Cruz Biotechnology (USA). Bcl-2, ERK42/44 and Par-4 antibodies were obtained from BD Bioscience, Santa Cruz Biotechnology (USA), Sigma and Cell Signaling Technology (CST). Phospho Akt (Ser473) and total Akt antibodies were purchased from Santa Cruz Biotechnology (USA). Species specific HRP-labeled and fluorescent labeled secondary antibodies were procured from Bio-rad (USA) and Molecular probes (USA) respectively.

### Cell Culture

Human Neural Glial cell-line (HNGC-2) was generated from human GBM tumor in National Centre for Cell Science (NCCS), Pune. HNGC-2 cell line is tumorigenic and forms neurospheres. These cells stain intensely for the neural stem cell markers-CD133, -nestin,bmi-1, musashi1, Sox2, and nucleostemin confirming the stem cell characters [10].

The cells were grown in DMEM medium supplemented with 5% fetal bovine serum (FBS, Gibco) and antibiotics (100 U/ml penicillin and 100 µg/ml streptomycin, Sigma, USA) in 5% CO_2_ humidified incubator at 37°C. Cells were seeded at a density of 0.3×10^6^ cells/ml in 6-well plates or 1×10^4^ cells/100 µl in 96-well plates and cultured for 24 h before treatment.

GBM tumor samples were provided by D.Y. Patil Medical College and Hospital (Pune). Grading of tumors was done according to WHO criteria by neuropathologist. The tissue was finely chopped or enzyme dissociated and plated in complete DMEM medium with antibiotics and serum in petri dishes. Primary cultures were established and maintained in DMEM medium supplemented with 10% FBS and antibiotics. Primary culture derived from GBM tumors (G1) was established and experiments were conducted with cells grown between passages 15–30.

### MTT assay

The effect of drugs on viability of HNGC-2 cells and G1 cells was determined by the standard colorimetric 3-(4, 5-dimethyl-2-thiazolyl)-2, 5-diphenyl-2H-tetrazolium bromide (MTT) assay. The drugs were solubilized in DMSO. Cells were treated for 24 h with varying doses of drugs or with DMSO, used as vehicle control. MTT (5 mg/ml) was added and formazan crystals formed were dissolved in 10% SDS along with 0.01N HCl. The absorbance was measured at 570 nm with reference to 640 nm using microplate reader (Spectromax 250, Molecular Devices). The number of live cells is directly proportional to formazan crystals formed. The percent viability was calculated considering values in vehicle control as 100%.

### Annexin V and PI staining

HNGC-2 cells were treated with tamoxifen (10µg/ml) for 12 h and apoptosis was determined using Annexin-V apoptosis detection kit (BD Pharmingen) according to the manufacturer's protocol. Analysis was performed by flow cytometry (FACS CantoII, Becton-Dickinson) using software Cell Quest Pro (Becton-Dickinson).

### Caspase-3 activity assay

Caspase-3 activity was determined using QIA91 Caspase 3 detection kit (Calbiochem, EMD chemicals, USA). After treatment, cells were processed according to the manufacturer's protocol and analyzed by flow cytometry using FL-1 channel on BD-FACS Caliber.

### Tunel assay

Tunel assay was performed by using Fluorescein FragEL™ DNA Fragmentation Detection Kit (Calbiochem Cat. No. QIA39) which labels DNA strand breaks by terminal deoxynucleotidyl transferase (TdT) enzyme tagged with fluorescein. After treatment, cells were processed and labeled with TdT Enzyme according to the manufacturer's protocol and analyzed by Flow cytometry using FL-1 as parameter on BD-FACS Caliber.

### Mitochondrial transmembrane potential (MMP)

The mitochondrial membrane potential was measured using Mitocapture Apoptosis Detection Kit (Oncogene Research). Cells treated for different time intervals were harvested and processed according to manufacturer's protocol. Flowcytometric analysis for disruption of mitochondrial membrane potential was done using FL-1 parameter in BD-FACS CantoII. Increase in green fluorescence depicts breakdown in mitochondrial membrane potential.

### Flow Cytometry Analysis of Bcl-2

HNGC-2 cells were analyzed to detect protein expression levels of Bcl-2 after treatment with tamoxifen for 24 h. Treated and untreated cells were harvested by trypsinization. Cells were fixed by paraformaldehyde and permeabilized with 0.2% triton X-100. Blocking was done using 3% BSA for 1 h. Cells were stained using primary antibody to Bcl-2 or respective isocontrol for 2 h at 4°C and then probed with anti-mouse oregon green. Analysis was performed on BD-FACS CantoII.

### Confocal laser scanning microscopy

Control and tamoxifen (10µg/mL) treated HNGC-2 and G1 cells, were fixed with 3.7% paraformaldehyde for 10 min and permeabilized for 5 min with 0.2% triton X-100. After blocking for 1 h in 3% BSA, cells were incubated with primary antibodies to Par-4, GRP78, and neural stem cells markers - Nestin and Bmi-1 (Chemicon), Sox-2 and mushashi (R&D Systems) and vimentin (Thermo Shandon Immunon Detection Systems) for 2 h followed by goat anti-rabbit Cy3 labeled antibody and phallodin (actin), (Molecular Probes, Invitrogen) for 60 min at room temperature. Nuclear staining was done by DAPI. Images were acquired using confocal laser scanning microscope (Carl Zeiss or Leica, Germany).

### Western blotting

Cells were harvested and lysed using RIPA lysis buffer (120 mM NaCl, 1.0% Triton X-100, 20 mM Tris–HCl, pH 7.5, 100% glycerol, 2 mM EDTA and protease inhibitor cocktail, Roche, Germany). Total protein (30µg) was electrophoresed on 10% SDS-polyacrylamide gels and blotted onto PVDF membrane (Millipore, Bedford, MA, USA). After blocking with 5% BSA at room temperature, the blots were probed with antibodies to Par-4, PARP and GRP78 overnight at 4°C. The bands were visualized by chemiluminescence using Super Signal West Femto Maximum Sensitivity Substrate (Pierce, USA). GAPDH and actin were used as loading controls for whole cell and cytoplasmic extracts and lamin A/C for nuclear extracts.

### Real time PCR

RNA isolation was performed using TRIzol reagent (Invitrogen, USA) and reverse-transcribed into cDNA (Promega) according to the manufacturer's instructions. Quantitative real time PCR was performed using SYBR Green Supermix (Biorad) in Realplex Real-Time Thermal Cycler (Eppendorf). The profile of thermal cycling consisted of initial denaturation at 95°C for 2 min, and 40 cycles at 95°C for 15 s and 60°C for 45 s for primer annealing and extension. Melting curve analysis was used to determine the specific PCR products. All primers used for Real-Time PCR analysis were synthesized by Integrated DNA Technologies, India. 18 s ribosomal RNA was used as an internal control. The sequence of the primers used was (5′ to 3′): Par-4: forward- GCAGATCGAGAAGAGGAAGC, reverse - GCAGATAGGAACTGCCTGGA, 18 s rRNA forward - AAACGGCTACCACATCCAAG, reverse – CCTCCAATGGATCCTCGTTA. The changes in the threshold cycle (C_T_) values were calculated by the equation: - ΔC_T_  =  C_T (target) −_ C_T (endogenous control)_ and fold difference was calculated as 2^−Δ (ΔC^_T_^)^.

### Transfections

HNGC-2 cells and G1 cells cultured in 6 well plate, at 70% confluency were transfected with Lipofectamine 2000 (Invitrogen) followed by treatment with ON-TARGET plus SMART pool, Human PAWR (5074) L-004434-00-0020 siRNA of Par-4 or non-targeting siRNA (Dharmacon, Inc.). After 48 h, the cells were treated with tamoxifen (10µg/mL) and analyzed for cell viability by MTT assay, flow cytometry for apoptosis and cell lysates were processed for western blotting.

For overexpression studies, cells were transfected with Par-4-GFP or with GFP plasmids. The plasmid containing the PAR-4 gene was a kind gift of Dr. Vivek Rangnekar, University of Kentucky. The efficiency of transfection was detected by immunostaining of Par-4 and after 24 h the cell viability was analyzed by MTT assay.

### Statistical Analysis

The data were represented as mean ± SD/SE and analyzed for Student's *t* test. p<0.05 values were assigned significance.

## Results

### HNGC-2 cells are sensitive to tamoxifen-induced cytotoxicity

**Figure 1:**
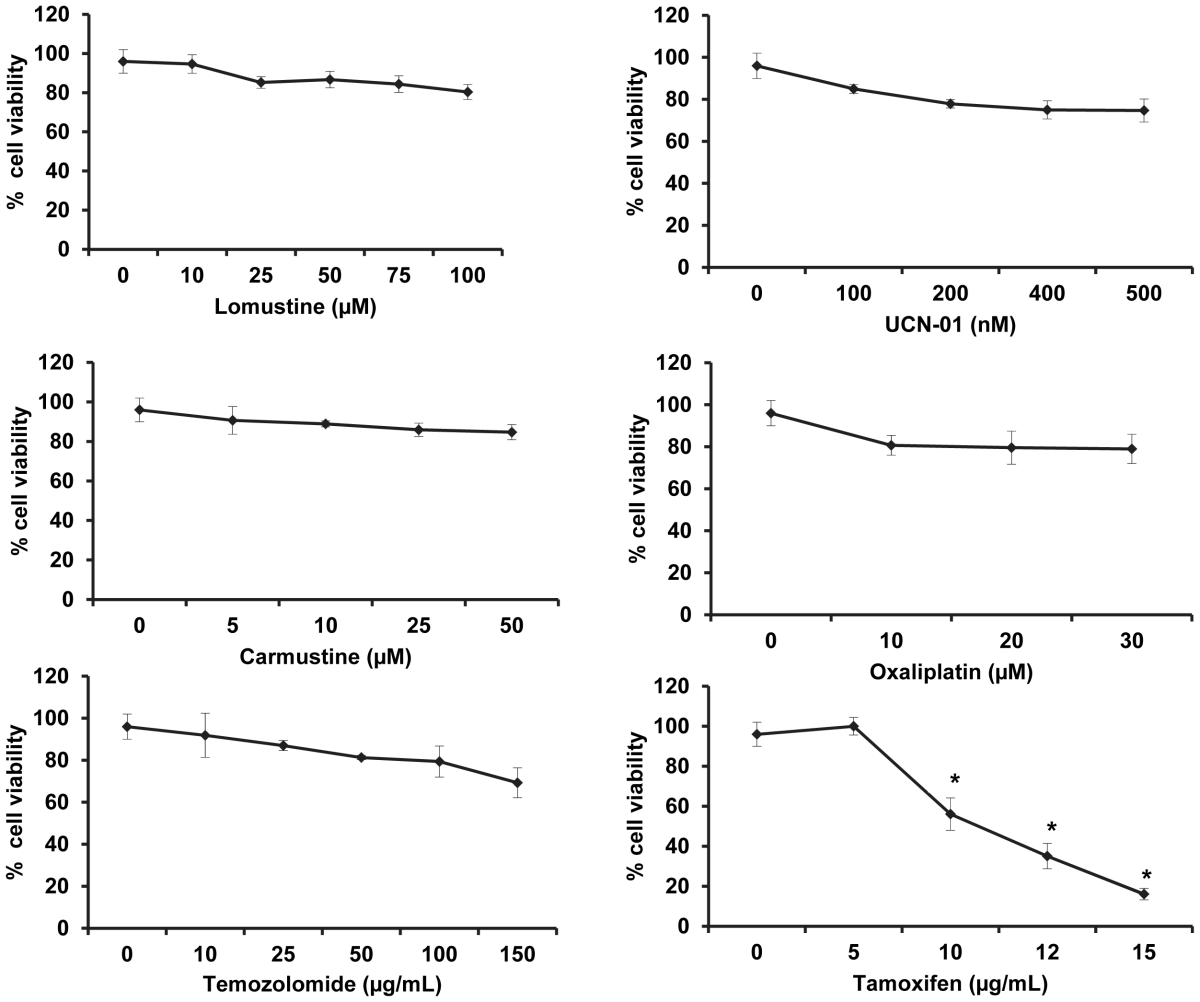
Dose dependent response to different drugs on cell viability. HNGC-2 cells were treated for 24 h with lomustine, carmustine, UCN-01, oxaliplatin, temozolomide, and tamoxifen (TAM) and cell viability was assessed by MTT assay. The cell viability of vehicle control cells was assumed as 100%. The data represents the mean ± SE (n = 3). * p< 0.01, comparison between vehicle control and treated cells.

HNGC-2 cells were treated with increasing doses of lomustine, carmustine, UCN-01, oxaliplatin, temozolomide, and tamoxifen (TAM) for 24 h and cell viability was measured by MTT assay. A significant decrease in cell viability was observed with TAM while other drugs were ineffective in inducing cell death in HNGC-2 cells (Fig. 1).

### Tamoxifen induces apoptosis in HNGC-2 cells

**Figure 2:**
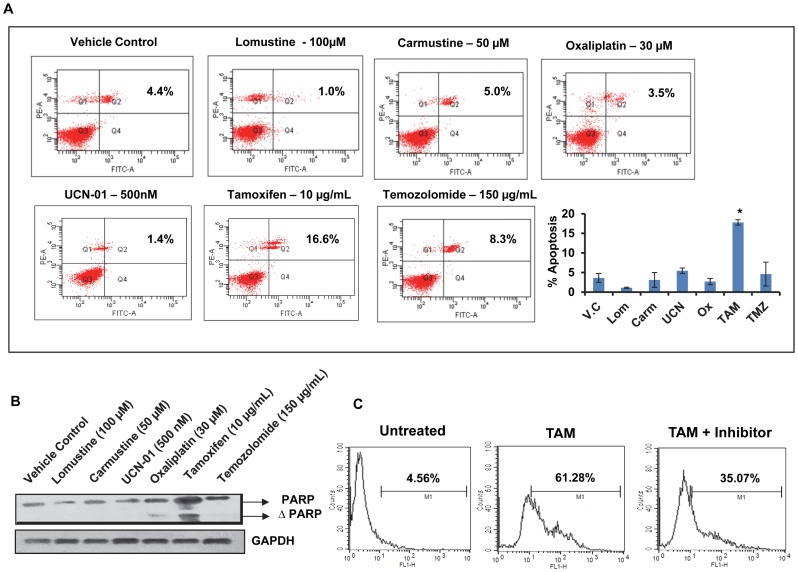
Tamoxifen induces cell death by apoptosis. (A) HNGC-2 cells were exposed to lomustine (Lom), carmustine (Carm), UCN-01, oxaliplatin (Ox), tamoxifen (TAM) and temozolomide (TMZ) for 12 h and stained by Annexin V (FL-1) and Propidium Iodide (FL-2) to perform flowcytometry analysis. The graph represents percent apoptotic population (Annexin V and PI positive). The bars represent mean± SE of three independent experiments. *p<0.05, treated vs. vehicle control (V.C). (B)Western blot depicts total and cleaved PARP in HNGC-2 cells treated with panel of drugs for 24 h, GAPDH was used as loading control. (C) HNGC-2 cells were treated with TAM for 24 h in the presence or absence of caspase-3 inhibitor (ZVAD-FMK), stained with FITC-DEVD-FMK and analyzed for caspase-3 activity. The values in the graphs are percent positive cells for caspase-3 activity. Histogram is representative data of two similar experiments.

To investigate whether the reduction in cell viability triggered by TAM was associated with apoptosis, dual staining with Annexin- V-FITC and propidium iodide was carried out. As shown in Fig. 2A, TAM induced significant apoptosis in comparison to other drugs used in the panel. Vehicle control cells showed 95% cell viability. For all further experiments untreated cell population was used as control. Western blotting analysis using antibody detecting total and cleaved PARP revealed that cell lysates from TAM-treated cells indicated increased band intensity corresponding to cleaved PARP. In the lysates of cells exposed to oxaliplatin a weak signal to cleaved PARP was observed (Fig. 2B). We further examined whether caspase-3 activity is involved in TAM induced-apoptosis using flowcytometric assay. We found that 61.28% of the cells treated with TAM for 24 h were positive for caspase-3 activity compared to 4.56% in untreated cells while caspase-3 inhibitor (ZVAD-FMK) reduced this population to 35.07% in TAM treated cells (Fig. 2C). These preliminary results suggested that HNGC-2 is sensitive to TAM-induced cell death that involved activation of caspase-3.

### Tamoxifen-induced apoptosis is associated with upregulation of Par-4

**Figure 3:**
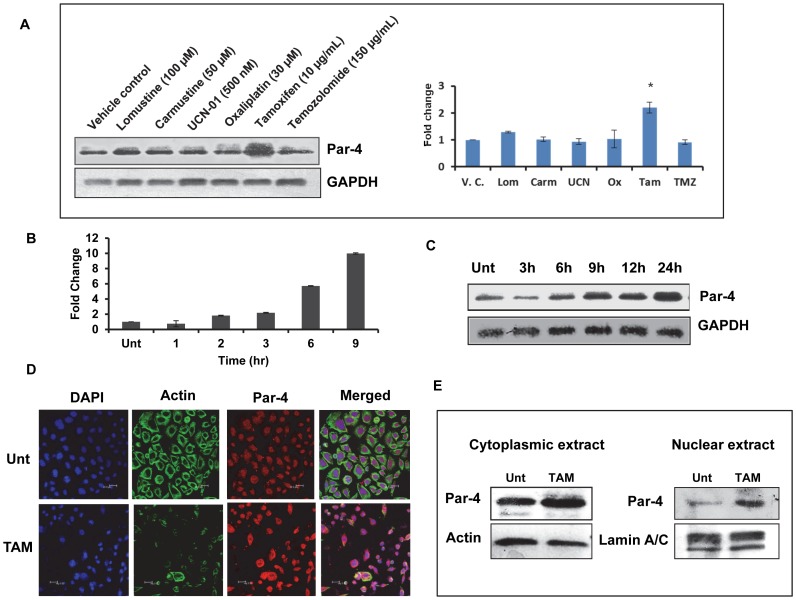
Expression of Par-4 in HNGC-2 cells treated with a panel of drugs. (A) Expression of Par-4 was determined by immunoblotting in HNGC-2 cells exposed to lomustine, carmustine, UCN-01, oxaliplatin, tamoxifen (TAM) and temozolomide for 24 h. The bars represent mean ± SE of three independent experiments. *p<0.05, treated vs. vehicle control. (B) Time dependent induction of Par-4 in HNGC-2 cells treated with TAM (10µg/mL). Fold induction of Par-4 mRNA level normalized using 18S rRNA, was assessed by quantitative real-time PCR. The data are expressed as the mean ± SD and are representative of two independent experiments that yielded similar results. (C) Par-4 protein levels relative to GAPDH were analyzed by western blot. (D) Par-4 expression (red) was visualized by probing with Cy3 antibody along with actin as green (Scale bar - 20µm). (E) Cell fraction lysates were used to determine Par-4 upregulation in cytoplasm and nucleus of HNGC-2 cells exposed to TAM (10 µg/mL), Actin and lamin A/C were used as loading controls respectively.

Prostrate apoptosis response-4 (Par-4) is a proapoptotic protein and overexpression of Par-4 sensitizes cancer cell lines and tumor cells to cytotoxicity triggered by anticancer drugs [18]. We therefore examined the relationship between the expression of Par-4 and susceptibility to TAM-induced apoptosis. HNGC-2 cells were treated with the same panel of drugs used in viability studies and Par-4 level was determined by immunoblotting. As depicted in Fig. 3A, TAM alone was effective in upregulating Par-4 significantly. Time kinetic experiments showed increase in transcript levels of Par-4 by tenfold on exposure to TAM (Fig. 3B). At the protein level, a marginal increase in Par-4 level was observed in cells treated with TAM for 6 h compared to control cells and the expression was significantly increased at later time points (Fig. 3C). Functional and localization studies have suggested that Par-4 localizes in the nucleus in most cancer cell lines and nuclear entry is essential for direct apoptosis [26], [27]. In this context, our data with confocal microscopy and western blot showed that exposure to TAM resulted in upregulation of Par-4 in nucleus as well as cytoplasm in HNGC-2 cells (Fig. 3D and E).

### Silencing of Par-4 protects cells from tamoxifen-induced apoptosis

**Figure 4:**
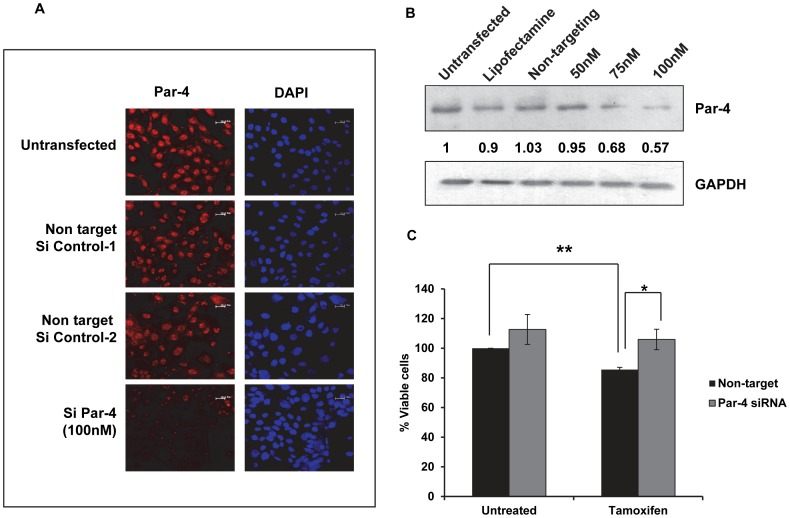
Silencing of Par-4 protects cells from Tamoxifen-induced cell death. (A) HNGC-2 cells were transfected with serial concentrations of Par-4 siRNA or control siRNA and analyzed for Par-4 expression by Immunofluorescence (Scale bar - 20µm) and (B) Immunoblotting. (C) Cells transfected with siRNA (100 nM) against Par-4 or with control siRNA (Nontarget) were treated with TAM (10µg/mL) for 24 h and cell viability was measured by MTT assay. The viability in control cells was assumed as 100%. The data represents the mean ± SE (n = 3). * p< 0.007, ** p< 0.002.

**Figure 5:**
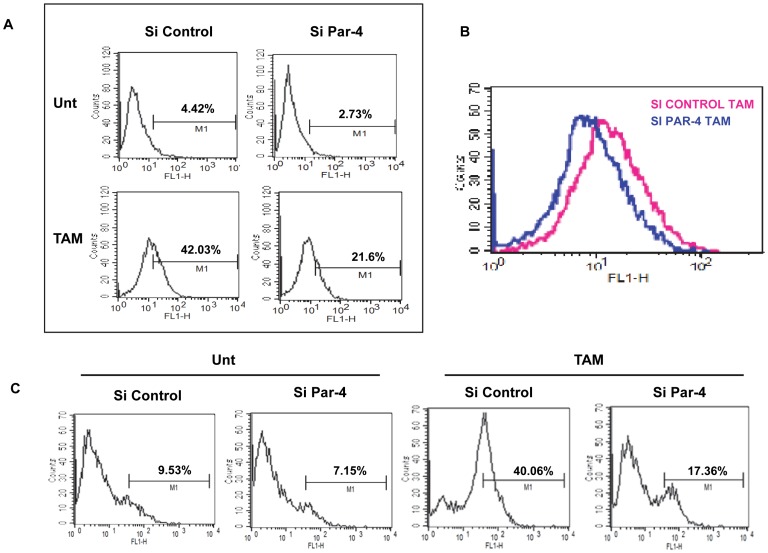
Effect of Par-4 silencing on apoptosis induced by Tamoxifen. HNGC-2 cells transfected with control siRNA (Nontarget) or siRNA (100 nM) against Par-4 were treated with Tamoxifen (10µg/mL) for 24 h and flowcytometry analysis was performed to confirm apoptosis by (A)TUNEL assay, (B) Represents the overlay profiles of HNGC-2 cells treated with transfected Si control (pink) and Par-4 siRNA (blue), (C) capase-3 activity. The numerical values in the plots are percent cells positive for apoptosis in (A) and caspase-3 activity in (C). The data is representative of two similar experiments.

Based on the result that apoptosis in HNGC-2 cells was associated with upregulation of Par-4, we conducted experiments to elucidate the function of Par-4 in TAM-induced apoptosis. HNGC-2 cells were transfected with control siRNA non target #1 or control siRNA non target #2 for negative control or Par-4 siRNA of serial concentrations (50, 75 and 100 nM) and the efficiency of silencing were tested by determining the expression of Par-4. A marked decrease in number of Par-4-expressing cells was detected by immunofluorescence and reduction in Par-4 levels by western blotting was observed in cells transfected with Par-4 siRNA but not with control siRNA (Fig. 4A and B). Further studies were performed using siRNA-control #2 and Par-4 siRNA at 100 nM. The transfected cells were exposed to TAM (10µg/mL) for 24 h and cell viability was measured by MTT assay. As depicted in Fig. 4C, Par-4 silenced cells showed higher cell viability in TAM-treated cells compared with control- non-target siRNA. Furthermore, the involvement of Par-4 in apoptosis was confirmed by TUNEL assay. In line with the MTT assay, exposure of Par-4 siRNA cells to TAM showed 21.6% TUNEL positivity in comparison with control siRNA (42.03%) resulting in 50% protection (Fig. 5A and B). Since our results suggested that caspase-3 is crucial for apoptosis induced by TAM, we assessed the effect of Par-4 expression on caspase-3 activity and as depicted in Fig. 5C, the loss of expression of Par-4 reduced the caspase-3 positive cells to 17.36% compared to control siRNA (40.06%). These results clearly indicated the crucial role of Par-4 in TAM-induced apoptosis that is mediated by caspase-3.

The cells treated with TAM showed a significant break down in mitochondrial membrane potential in time dependent manner implicating the role of mitochondria in apoptosis (Fig. S1).

Par-4 is reported to function as proapoptotic molecule by negatively regulating Bcl-2 [28], [29] and precedes loss of mitochondrial membrane potential [28]. In our study, flow cytometry analysis revealed that the percent positivity for Bcl-2 in cells treated TAM for 24 h was 43.4% compared to 68.6% in control. The MFI was also dramatically reduced in treated cells (Fig. S2). Interestingly, silencing of Par-4 did not affect the expression of Bcl-2 in HNGC-2 cells suggesting that Par-4 may not be directly involved in its regulation (Fig. S3).

### Regulation of Akt and ERK signaling by tamoxifen and Par-4

**Figure 6:**
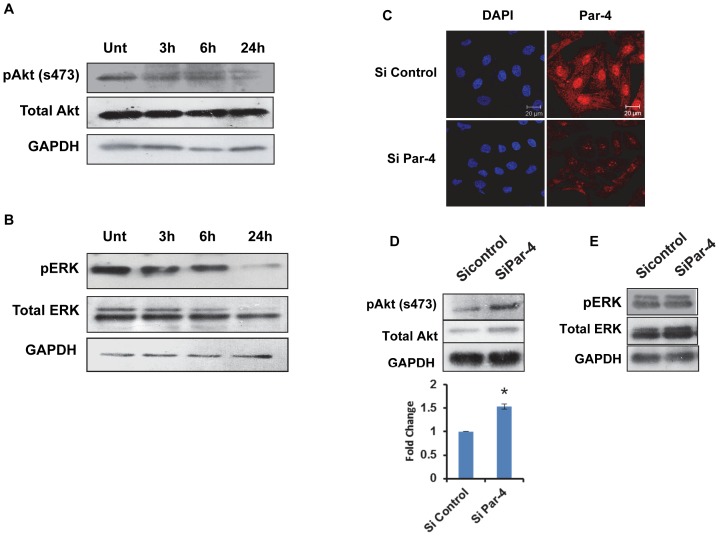
Tamoxifen induced Par-4 regulates Akt and ERK signaling in HNGC-2 cells. HNGC-2 cells treated with TAM (10 µg/mL) for increasing time periods were analyzed for the expression pattern of (A) pAkt (Ser473),total Akt and (B) pERK (42/44) and total ERK by western blotting. GAPDH was used as loading control. (C) HNGC-2 cells were transfected with control siRNA and Par-4 siRNA (100 nM) and analyzed for expression of Par-4 by immunofluorescence. (D) Transfected control and Par-4 silenced cells were analyzed for phosphoAkt (Ser473) total Akt and (E) phosphoERK and total ERK signaling by western blotting. Fold change is shown by bars, mean ± SD of three independent experiments. *p<0.05, control siRNA vs. Par-4 siRNA cells.

We next studied the effect of TAM in regulation of signaling pathways involving Akt and ERK 42/44, in HNGC-2 cells. The western blot analysis revealed that TAM significantly decreased the activation of Akt (ser473) and ERK42/44 in a time dependent manner suggesting their involvement in tamoxifen-induced apoptosis (Fig. 6A and B). In this regard, as TAM increased the expression of Par-4, it was of interest to examine whether Par-4 modulated the expression of Akt and ERK42/44. For this purpose, HNGC-2 cells were transfected with Par-4 specific siRNA or non-target siRNA and analysed for phosphorylated Akt and ERK42/44. As shown in Fig. 6D, the expression of phosphorylated Akt (ser473) was significantly (1.53 fold) enhanced in Par-4 knock down cells but loss of expression of Par-4 had no effect on the levels of phosphorylated and total Erk42/44 (Fig. 6E). These data suggested a role for Par-4 in Akt-mediated signalling but not in activation of ERK. However, based on the kinetics of expression of Par-4, phosphorylated Akt (Ser 473) and ERK42/44, it may be concluded that cellular mechanisms other than those involved in the downstream effects of PAR-4 may also contribute to Tamoxifen-induced PAR-4 activation.

### TAM- induced cell death in primary cultures derived from GBM tumor involves Par-4

Since HNGC-2 cell line is well established and has gone through course of 100 passages, it was of relevance to accentuate applicability of role of Par-4 in apoptosis. To this end, we specifically selected primary cultures derived from GBM tumors (G1) that are positive for neural stem cell markers. In comparison with HNGC-2 cells that expressed all the markers abundantly, G1 cell cultures stained intensely for musashi, nestin and vimentin but faintly for sox2 and Bmi-1. Interestingly, on exposure to TAM, the expression of stem markers were significantly reduced in both the cell types (Fig. S4 and S5).

**Figure 7:**
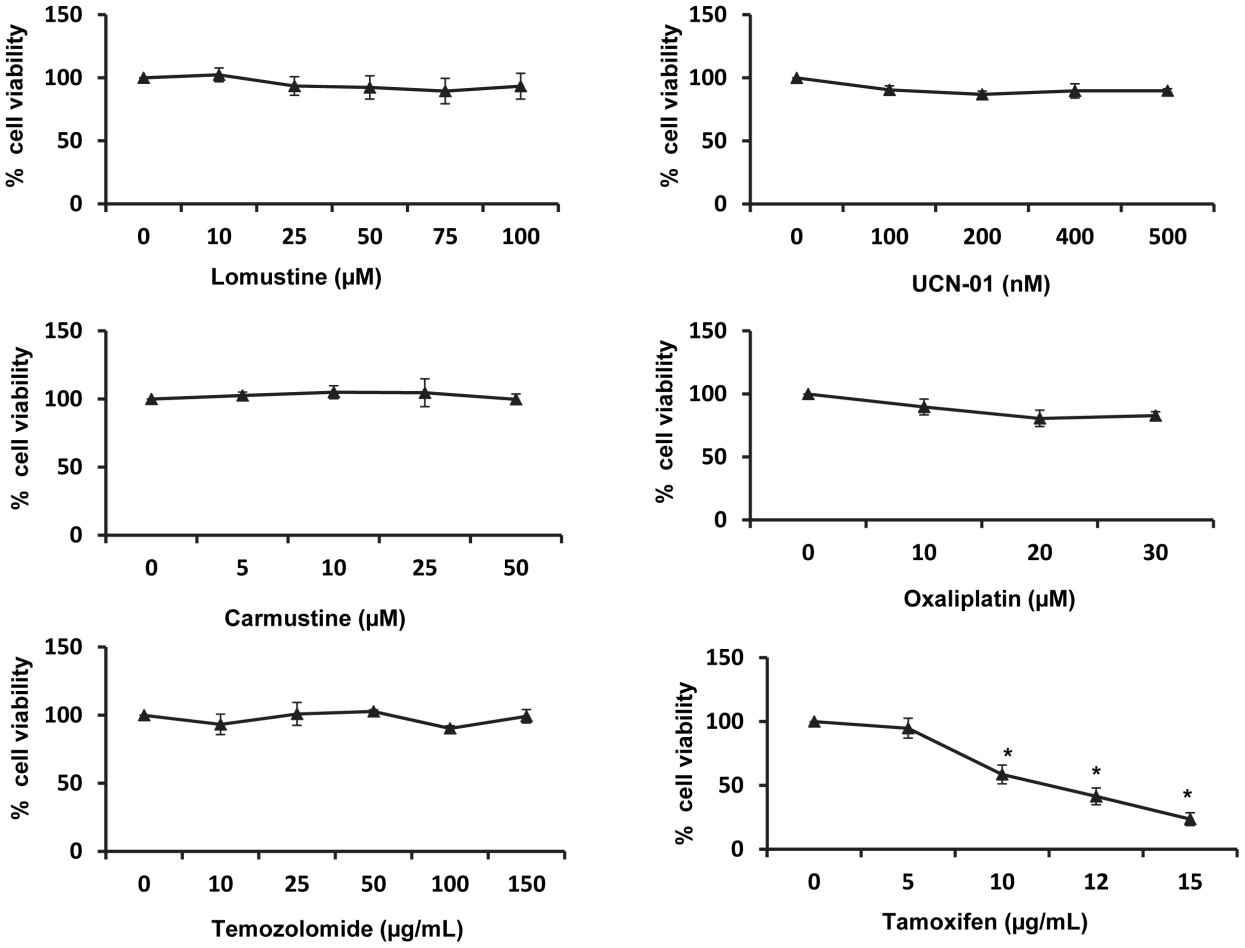
Dose dependent response of primary cultures to different drugs. Primary GBM (G1) cells were treated with lomustine, carmustine, UCN-01, oxaliplatin, temozolomide, and tamoxifen (TAM) for 24 h and cell viability was assessed by MTT assay. The viability in untreated control cells was assumed as 100%. The data represents the mean ± SE (n = 3). * p< 0.01 difference between untreated control vs. TAM treated cells.

**Figure 8:**
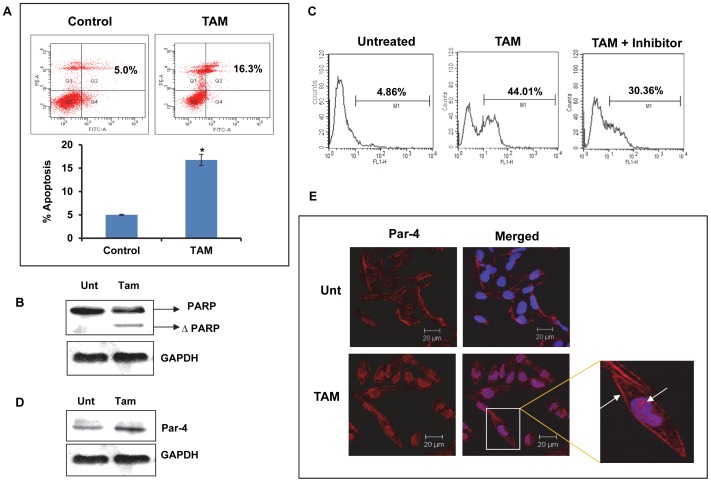
Tamoxifen induces apoptosis and Par-4 expression in Primary GBM cells. (A) G1 cells were treated with TAM for 12 h and analyzed for apoptosis by staining cells with Annexin V and PI followed by flow cytometry analysis. The bars in the histogram represent mean ±SE of three independent experiments. * p<0.05- treated vs. untreated control. (B) Cells were exposed to TAM for 24 h and analyzed for PARP by immunoblotting with GAPDH as loading control. (C) Caspase-3 activity analyzed by flow cytometry in control and treated cells with TAM for 24 h in the presence or absence of caspase-3 inhibitor (ZVAD-FMK). The numerical values are percent positive cells for caspase-3 activity. (D) Expression of Par-4 in TAM treated G1 cells depicted by immunoblotting with GAPDH as loading control. (E) Immunofluorescence shows the expression of Par-4 in G1 cells treated with TAM. The cells stained with Cy-3 (red) represents the expression and localization of Par-4 in untreated and treated cells (Scale bar - 20µm).

Consistent with our data in HNGC-2 cells, we found that of the panel of drugs used, G1 cells were sensitive to TAM alone (Fig. 7). Dual staining with Annexin- V-FITC and propidium iodide revealed 16.3% positivity in TAM-treated cells compared to 5% positivity in control cells (Fig. 8A). Analogous to this data, a distinct band of cleaved PARP was observed in treated cells by western blot analysis (Fig. 8B). Flowcytometry analysis illustrated (Fig. 8C) that caspase-3 activity in the TAM-treated cells was reduced by 13.65% in the presence of caspase-3 inhibitor confirming the involvement of caspase-3. TAM enhanced the expression of Par-4 as visualized by western blotting and confocal microscopy (Fig. 8D and E).

**Figure 9:**
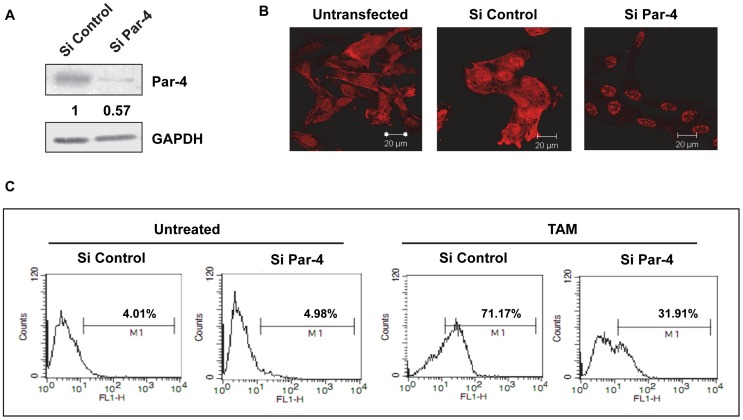
Effect of Par-4 silencing on apoptosis in primary GBM cells treated with Tamoxifen. (A) G1 cells were transfected with control siRNA or Par-4 siRNA (100 nM) and analyzed for its expression by immunoblotting and (B) Immunofluorescence (Scale bar - 20µm). (C) The control and Par-4 transfected cells were exposed to TAM (15µg/ml) for 36 h and assessed for caspase-3 activity. Values in the plot indicate percent cells positive for caspase-3 activity.

To study the role of Par-4 in TAM-induced apoptosis, G1 cells transfected with Par-4 siRNA or control siRNA were treated with tamoxifen and caspase-3 activity was measured as a read out for apoptosis. The efficacy of Par-4 silencing was confirmed with significant decrease in Par-4 levels in cells transfected with Par-4 siRNA compared to control siRNA and untransfected cells (Fig. 9A and B). We also examined the influence of Par-4 silencing on exposure of cells to TAM. The caspase-3 activity was markedly increased in treated cells (71.17%) compared to untreated cells demonstrating apoptosis-inducing effect of TAM. The reduction of caspase-3 positivity in TAM treated Par-4 siRNA cells (31.91%) confirmed the protection conferred by Par-4 (Fig. 9C).

### Role of secretory Par-4 and GRP78 in TAM-induced apoptosis

**Figure 10:**
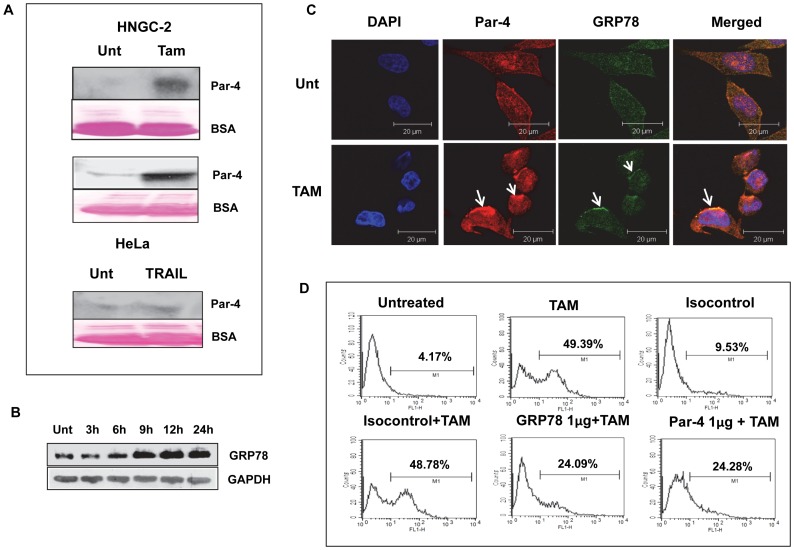
Role of secretory Par-4 in TAM induced apoptosis. (A) HNGC-2 cells were treated with TAM for 24 h and extracellular Par-4 was analyzed by western blotting in conditioned medium (neat -upper blot) and after concentrating by 30 KD cut-off filters (lower blot). The adjacent blots show ponceau staining of BSA, used as loading control. Expression of Par-4 in conditioned medium of HeLa cells treated with TRAIL was used as positive control for secretory Par-4. (B)Time dependent induction of GRP78 in HNGC-2 cells treated with TAM (10 µg/mL) was determined by western blotting. GAPDH was used as a loading control. (C) Par-4(red) localization with GRP78 (green) on cell membrane in TAM treated HNGC-2 cells (Scale bar - 20µm). (D) HNGC-2 cells were exposed to GRP78 and Par-4 antibody for 1 h prior to TAM treatment and apoptosis was assessed for caspase-3 activity by flow cytometry analysis. IgG was used as isotype control. Values in plots depicts percent cells positive for caspase-3 activity.

To attain insight into the mechanism of action of Par-4 during TAM-induced apoptosis, we also explored the role of extrinsic Par-4 in the process. To address this point,conditioned medium was collected from TAM treated HNGC-2 cells after 24 h. Western blotting was done using conditioned medium (neat) and in medium concentrated using 30 kDa cut-off filters. We found that Par-4 was abundantly secreted in treated cells as evident by its presence in medium. While the concentrated conditioned medium from control cells displayed a faint band corresponding to Par-4, the medium from treated cells showed robust band distinctly. In these experiments, spent medium from HeLa cells treated with TRAIL were used as positive controls (Fig. 10A). Extracellular Par-4 is reported to induce apoptosis by binding to the stress response protein, glucose-regulated protein-78 (GRP78) [21]. We therefore hypothesized that TAM-mediated apoptosis in HNGC-2 cells might involve GRP78. The expression of GRP78 was significantly increased on exposure to TAM up to 9 h of treatment and the level was maintained till 24 h (Fig. 10B). To visualize the distribution of GRP78, confocal microscopy was performed. As depicted in Figure 10C, GRP78 was localized in the membrane of cells treated with TAM and overlapped with Par-4 suggesting co-localization of the two proteins. Furthermore, the localization of GRP78 to membrane was inhibited in cells transfected with si-Par-4 but not control siRNA indicating that intracellular Par-4 is crucial for membrane localization of GRP78 (Fig. S6). To confirm the role of Par-4 and GRP78 in TAM-induced apoptosis, HNGC-2 cells were exposed to GRP78 or Par-4 antibody for 1 h prior to treatment with TAM and caspase-3 activity was measured after 24 h. Flow cytometry analysis revealed that antibodies to GRP78 and Par-4 reduced the caspase-3 positive cells from 49.39% with treatment to TAM to 24.09 and 24.28% respectively; the isotype control antibody had no effect on the capase-3 activity (Fig. 10D). Collectively, these results suggest that the mechanism of TAM-induced apoptosis in HNGC-2 cells involves extracellular Par-4 and GRP78.

### Role of intracellular Par-4 in TAM induced cell death in HNGC-2 cells

**Figure 11:**
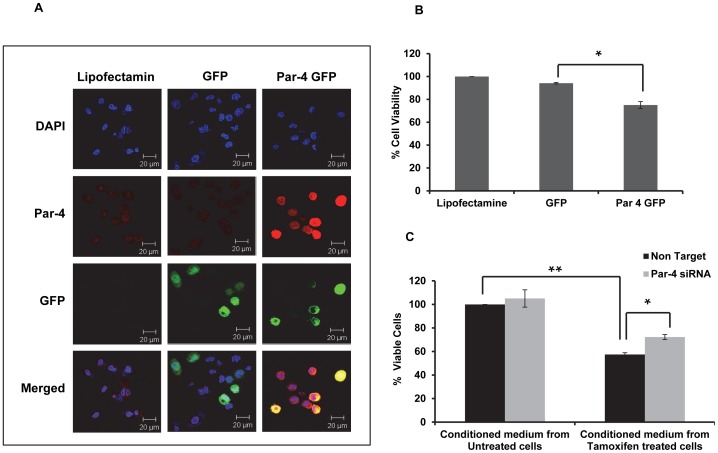
Intracellular Par-4 induces cell death in HNGC-2 cells. (A) HNGC-2 cells were transiently transfected with Par-4 plasmid tagged with GFP and GFP alone and analyzed for overexpression of Par-4 by Immunofluorescence (Scale bar - 20µm). (B) Par-4 overexpressed cells were assessed for cell viability after 24 h by MTT assay. The bars in the histogram represent mean± SD of three independent experiments, *p<0.05. (C) Cells transfected with siRNA (100 nM) against Par-4 or with control siRNA (Nontarget) were treated with conditioned medium containing secretory Par-4 for 24 h and cell viability was measured by MTT assay. The viability in control cells was assumed as 100%. The data represents the mean ± SE (n = 3). * p< 0.05, **p< 0.005.

To elucidate the role of Par-4 in TAM-induced apoptosis, HNGC-2 cells were transiently transfected with Par-4-GFP plasmid (Fig. 11A) and after 24 h viability was assessed by MTT assay (Fig. 11B). Par-4 overexpressing cells showed 25% cell death compared to control GFP transfected cells (5.83%) suggesting that overexpression of Par-4 is enough to induce cell death in HNGC-2 cells. To further confirm the contribution of intracellular Par-4, condition medium of TAM-treated cells (containing secretory Par-4) was added to control and si-Par-4 transfected HNGC-2 cells and analyzed for cell viability. As depicted in Fig. 11C, we observed a significant decrease in cell viability of control transfected cells (57.48%) in comparison to Si-Par-4 transfected cells (72.3%) suggesting that intracellular Par-4 is essential for the effect induced by conditioned medium of TAM-treated cells.

## Discussion

In this study, we investigated the role of Par-4 in cellular responses to chemotherapeutic drugs in cancer stem cells (CSC). For this purpose, we used two culture systems derived from GBM tumors - an established cell line - HNGC-2 and primary cultures (G1) expressing neural stem cell markers. To study the effect, the cells were exposed to a panel of alkylating agents that are reported to be effective for treatment of gliomas which includes - lomustine, carmustine, UCN-01, oxaliplatin, temozolomide and tamoxifen, [30]–[34]. Among these drugs, only tamoxifen (TAM) induced apoptosis in both the cell types and upregulated the expression of Par-4 suggesting an association of Par-4 and sensitivity to TAM treatment.

A direct correlation of Par-4 and apoptosis induced by various drugs has been demonstrated in cancers of colon and pancreas [19], [26]. Consistent with these reports, we found that Par-4 specific siRNA effectively inhibited apoptosis confirming the involvement of Par-4 in TAM-induced apoptosis. Ectopic expression of Par-4 in renal cancer cells sensitizes TRAIL-resistant Caki cells to apoptosis associated with activation of caspase-8, caspase-3 and modulation of DR5, Bcl-2, Akt, and NF-κB [18]. In CNS tumor cells including gliomas, ectopic expression of Par-4 was shown to be sufficient to induce apoptosis, in contrast to other cancer cells where overexpression of Par-4 levels only renders them sensitive to apoptotic stimuli [35]. Our data in HNGC-2 cells suggest that overexpression of Par-4 is enough for inducing cell death. Cellular localization of Par-4 is variable in different cell types [36]. Nuclear localization of Par-4 is a prerequisite for apoptosis in most cancer cells and ectopically expressed Par-4 readily translocate to the nucleus to induce apoptosis [18], [36]. Exposure to TAM in HNGC-2 cells and G1 cells resulted in upregulation of Par-4 throughout the membrane, cytosol as well as nucleus in comparison with untreated cells.

The proapoptotic function of Par-4 in the nucleus affects the inhibitory action of NF-κB-mediated mechanisms leading to suppression of survival genes of Bcl-2 family and other antiapoptotic proteins [37]–[39]. At the cytoplasmic level, Par-4 inhibits the phosphorylation and translocation of NF-κB to nucleus in TNF-α stimulated response [20]. Other studies have demonstrated that intracellular Par-4 down-regulates Bcl-2 protein in neoplastic lymphocytes [40] and at transcript level in prostate cancer cell lines [41]. We observed a significant decrease in Bcl-2 positive cells and fluorescence intensity in HNGC-2 cells exposed to TAM. However, Bcl-2 expression was independent of Par-4 in HNGC-2 cells as the expression of Bcl-2 was not significantly different in control and si-Par-4 transfected cells. Our findings that TAM-induced apoptosis was associated with break down in mitochondrial membrane potential (MMP) confirmed by the earlier studies in breast cancer cells [42].

Caspase -3 dependent cleavage of Par-4 and the accumulation of the cleaved fragment in nucleus is shown to enhance the apoptotic activity in human normal and cancer cell lines during cisplatin-induced apoptosis [43] and in sphingosine-induced apoptosis in cancer cells [44]. In our study, we found that caspase-3 inhibitor (ZVAD-FMK) inhibited apoptosis and abrogation of Par-4 decreased caspase-3 activity. Furthermore, time kinetics experiments revealed that exposure to TAM resulted in time dependent increase in expression of Par-4 with highest level at 24 h. It is noteworthy that at this time point the caspase-3 activity was also significantly high; suggesting that it is unlikely to predict Par-4 is cleaved by caspase-3. However, we cannot rule out the possibility as we measured total but not cleaved Par-4. Thus our findings lead us to conclude that the mechanism involves activation of caspase-3, break down in MMP and reduction in Bcl-2.

Several studies have reported activation of Akt and ERK42/44 signaling pathways to be important in drug resistance [45], [46]. To this end, our data demonstrated that TAM reduced the activation of Akt. Also, silencing of Par-4 significantly upregulated the level of pAkt (Ser-473) indicating that Akt is under the regulation of Par-4. In pancreatic tumors, Par-4 is known to act as a negative regulator of Akt activation via PKC zeta [47], [48]. Given that PKC zeta is expressed highly in gliomas [49] and is associated with Par-4 [50], it is reasonable to speculate that in glioma stem cells, regulation of Akt by Par-4 involves modulation of PKC zeta. Reports on the effect of TAM on ERK42/44 signaling is contradictory, while ERK is activated in MCF-7 cell line [51], [52], other studies have demonstrated reduced levels of ERK42/44 in melanoma cells [52]. In HNGC2 cells, exposure to TAM reduced the levels of ERK42/44.

While the role of intrinsic Par-4 is well documented in different cancer cell types in response to a wide arrange of drugs and anticancer agents, recent study by Burikhanov *et al*., 2009 demonstrated that conditioned medium obtained from Par-4-GFP transfected cells induced apoptosis in hormone-independent prostate cancer PC-3 cells. Furthermore, the secretory Par-4 was shown to activate an extrinsic pathway involving cell surface GRP78 receptor and FADD/caspase-8/caspase-3 pathway for apoptosis [21]. A recent report revealed that NF-κB can downregulate trafficking of the PAR-4 receptor, GRP78 from the endoplasmic reticulum to the cell surface inhibiting the apoptosis activity of secretory Par-4 [53]. Besides, its function as a proapoptotic protein, secretory Par-4 is reported to regulate MMP-2-mediated motility in cervical and prostate cancer in response to 3-azido Withaferin A [54]. In most solid tumors, high glucose metabolism and hypoxic conditions result in high ER stress leading to upregulation in membrane-bound GRP78 expression [11], [55]. On the basis of these reports, we explored the possibility that TAM might induce extracellular Par-4 in HNGC-2. We found that levels of secretory Par-4 and expression of GRP78 were robustly increased in HNGC-2 cells treated with TAM compared to controls. Importantly, neutralization of Par-4 or blocking of GRP78 with specific antibodies inhibited TAM-induced apoptosis suggesting the role of these molecules in apoptosis. Our data also confirms the earlier report that intracellular Par-4 is necessary for secretory Par-4 [21]. Collectively, our findings conclude that both intracellular and secretory Par-4 is involved in TAM-induced apoptosis in cancer stem cell line of glioma origin.

A subtype of high grade gliomas are characterized by the presence of neural stem cell markers [56]. Many drugs including angiogenesis inhibitors, all-trans-retinoic acid are reported to reduce stemness properties of gliomas in addition to their cytotoxic effect [57]–[59] Though TAM is used in treatment for gliomas, there are no reports on the effect TAM on neural stem cell markers. It is noteworthy that TAM reduced the expression of all stem cell markers - Nestin, Bmi1, Vimentin, Sox2, and Musashi in HNGC-2 and G1 cells, implicating its potential as a stemness inhibiting drug. Thus, with the current chemotherapeutic modalities targeting CSC as the drug-resistant population, it would be interesting to screen potential anticancer compounds/agents for their ability to upregulate intracellular and secretory Par-4 in these cells and analyze their association with cellular cytotoxicity.

## Supporting Information

Figure S1 **Tamoxifen induced cell death through mitochondrial membrane rupture.** (A) HNGC-2 cells were treated by TAM for 3 h, 6 h and 12 h, stained with Mitocapture Reagent (Calbiochem) and analyzed for mitochondrial membrane potential by flowcytometry using FITC channel for green monomers, Ex/Em  =  488/530±30 nm (for details refer materials and methods). Y-axis represents percent positive cells for green fluorescence with respect to untreated controls. (B) Mitochondrial membrane potential of HNGC-2 cells, untreated and TAM treated at 6 h were analysed by flow cytometry same as above. Bars indicate mean ± SD (n = 3). * p< 0.018
(TIF)

Figure S2 **Tamoxifen downregulates Bcl-2 expression in HNGC-2 cells.** HNGC-2 cells were treated with TAM for 24 h and expression of Bcl-2 was assessed by Flow cytometry using FITC channel (for details refer materials and methods). Y-axis represents percent positive cells and mean fluorescence intensity (MFI) for green fluorescence with respect to isocontrol.
(TIF)

Figure S3 **Silencing of Par-4 does not affect Bcl-2 expression significantly in HNGC-2 cells.** HNGC-2 cells were transfected with control siRNA and Par-4 siRNAand further analyzed for Bcl-2 levels by flow cytometry (BD Caliber) using FL-1 channel for green fluorescence. Markers in the plots represent percent positive cells with respect to isocontrol.
(TIF)

Figure S4 **Effect of tamoxifen on stem cell markers in HNGC-2 cells.** HNGC-2 cells were treated with TAM and expression of stem cell markers like Bmi1, Nestin, Musashi, Sox2 and Vimentin were visualized using Cy3 secondary antibody (red) using Carl Zeiss/Leica, confocal Microscope (Scale bar - 20µm).
(TIF)

Figure S5 **Effect of tamoxifen on stem cell markers in Primary GBM cells (G1).** G1 cells were treated with TAM and expression of stem cell markers like Bmi1, Nestin, Musashi, Sox2 and Vimentin were visualized using Cy3 secondary antibody (red) using Carl Zeiss/Leica, confocal Microscope (Scale bar - 20µm).
(TIF)

Figure S6 **Translocation of GRP78 to the membrane depends on Par-4 levels in HNGC-2 cells.** Par-4 siRNA transfected cells were treated with tamoxifen and visualized for GRP78 (green) expression and localization by immunofluorescence (Scale bar - 20µm).
(TIF)

## References

[1] Demuth T, Rennert JL, Hoelzinger DB, Reavie LB, Nakada M, et al (2008) Glioma cells on the run - the migratory transcriptome of 10 human glioma cell lines. BMC Genomics 9: 54. 18230158 10.1186/1471-2164-9-54 PMC2275271

[2] Tait MJ, Petrik V, Loosemore A, Bell BA, Papadopoulos MC (2007) Survival of patients with glioblastoma multiforme has not improved between 1993 and 2004: analysis of 625 cases. Br J Neurosurg 21: 496–500. 17852105 10.1080/02688690701449251

[3] Reya T, Morrison SJ, Clarke MF, Weissman IL (2001) Stem cells, cancer, and cancer stem cells. Nature 414: 105–111. 11689955 10.1038/35102167

[4] Visvader JE, Lindeman GJ (2008) Cancer stem cells in solid tumours: accumulating evidence and unresolved questions. Nat Rev Cancer 8: 755–768. 18784658 10.1038/nrc2499

[5] Singh SK, Hawkins C, Clarke ID, Squire JA, Bayani J, et al (2004) Identification of human brain tumour initiating cells. Nature 432: 396–401. 15549107 10.1038/nature03128

[6] Lu C, Shervington A (2008) Chemoresistance in gliomas. Mol Cell Biochem 312: 71–80. 18259841 10.1007/s11010-008-9722-8

[7] Liu G, Yuan X, Zeng Z, Tunici P, Ng H, et al (2006) Analysis of gene expression and chemoresistance of CD133+ cancer stem cells in glioblastoma. Mol Cancer 5: 67. 17140455 10.1186/1476-4598-5-67 PMC1697823

[8] Beier D, Schulz JB, Beier CP (2011) Chemoresistance of glioblastoma cancer stem cells–much more complex than expected. Mol Cancer 10: 128. 21988793 10.1186/1476-4598-10-128 PMC3207925

[9] Shiras A, Bhosale A, Shepal V, Shukla R, Baburao VS, et al (2003) A unique model system for tumor progression in GBM comprising two developed human neuro-epithelial cell lines with differential transforming potential and coexpressing neuronal and glial markers. Neoplasia 5: 520–532. 14965445 10.1016/s1476-5586(03)80036-2 PMC1502577

[10] Shiras A, Chettiar ST, Shepal V, Rajendran G, Prasad GR, et al (2007) Spontaneous transformation of human adult nontumorigenic stem cells to cancer stem cells is driven by genomic instability in a human model of glioblastoma. Stem Cells 25: 1478–1489. 17332509 10.1634/stemcells.2006-0585

[11] Shrestha-Bhattarai T, Rangnekar VM (2010) Cancer-selective apoptotic effects of extracellular and intracellular Par-4. Oncogene 29: 3873–3880. 20440265 10.1038/onc.2010.141 PMC2900490

[12] Franchitto A, Torrice A, Semeraro R, Napoli C, Nuzzo G, et al (2010) Prostate apoptosis response-4 is expressed in normal cholangiocytes, is down-regulated in human cholangiocarcinoma, and promotes apoptosis of neoplastic cholangiocytes when induced pharmacologically. Am J Pathol 177: 1779–1790. 20724592 10.2353/ajpath.2010.091171 PMC2947274

[13] Irby RB, Kline CL (2013) Par-4 as a potential target for cancer therapy. Expert Opin Ther Targets 17: 77–87. 23062118 10.1517/14728222.2013.731047

[14] Cook J, Krishnan S, Ananth S, Sells SF, Shi Y, et al (1999) Decreased expression of the pro-apoptotic protein Par-4 in renal cell carcinoma. Oncogene 18: 1205–1208. 10022126 10.1038/sj.onc.1202416

[15] Moreno-Bueno G, Fernandez-Marcos PJ, Collado M, Tendero MJ, Rodriguez-Pinilla SM, et al (2007) Inactivation of the candidate tumor suppressor par-4 in endometrial cancer. Cancer Res 67: 1927–1934. 17332319 10.1158/0008-5472.CAN-06-2687

[16] Qiu G, Ahmed M, Sells SF, Mohiuddin M, Weinstein MH, et al (1999) Mutually exclusive expression patterns of Bcl-2 and Par-4 in human prostate tumors consistent with down-regulation of Bcl-2 by Par-4. Oncogene 18: 623–631. 9989812 10.1038/sj.onc.1202344

[17] Alvarez JV, Pan TC, Ruth J, Feng Y, Zhou A, et al (2013) Par-4 Downregulation Promotes Breast Cancer Recurrence by Preventing Multinucleation following Targeted Therapy. Cancer Cell 24: 30–44. 23770012 10.1016/j.ccr.2013.05.007 PMC3808871

[18] Lee TJ, Jang JH, Noh HJ, Park EJ, Choi KS, et al (2010) Overexpression of Par-4 sensitizes TRAIL-induced apoptosis via inactivation of NF-kappaB and Akt signaling pathways in renal cancer cells. J Cell Biochem 109: 885–895. 20127709 10.1002/jcb.22504

[19] Wang BD, Kline CL, Pastor DM, Olson TL, Frank B, et al (2010) Prostate apoptosis response protein 4 sensitizes human colon cancer cells to chemotherapeutic 5-FU through mediation of an NF kappaB and microRNA network. Mol Cancer 9: 98. 20433755 10.1186/1476-4598-9-98 PMC2883962

[20] Diaz-Meco MT, Lallena MJ, Monjas A, Frutos S, Moscat J (1999) Inactivation of the inhibitory kappaB protein kinase/nuclear factor kappaB pathway by Par-4 expression potentiates tumor necrosis factor alpha-induced apoptosis. J Biol Chem 274: 19606–19612. 10391896 10.1074/jbc.274.28.19606

[21] Burikhanov R, Zhao Y, Goswami A, Qiu S, Schwarze SR, et al (2009) The tumor suppressor Par-4 activates an extrinsic pathway for apoptosis. Cell 138: 377–388. 19632185 10.1016/j.cell.2009.05.022 PMC2774252

[22] Parney IF, Chang SM (2003) Current chemotherapy for glioblastoma. Cancer J 9: 149–156. 12952300 10.1097/00130404-200305000-00003

[23] Spence AM, Peterson RA, Scharnhorst JD, Silbergeld DL, Rostomily RC (2004) Phase II study of concurrent continuous Temozolomide (TMZ) and Tamoxifen (TMX) for recurrent malignant astrocytic gliomas. J Neurooncol 70: 91–95. 15527114 10.1023/b:neon.0000040837.68411.97

[24] Sabioni P, Baretta IP, Ninomiya EM, Gustafson L, Rodrigues AL, et al (2008) The antimanic-like effect of tamoxifen: Behavioural comparison with other PKC-inhibiting and antiestrogenic drugs. Prog Neuropsychopharmacol Biol Psychiatry 32: 1927–1931. 18930105 10.1016/j.pnpbp.2008.09.023

[25] Hui AM, Zhang W, Chen W, Xi D, Purow B, et al (2004) Agents with selective estrogen receptor (ER) modulator activity induce apoptosis in vitro and in vivo in ER-negative glioma cells. Cancer Res 64: 9115–9123. 15604281 10.1158/0008-5472.CAN-04-2740

[26] Azmi AS, Wang Z, Burikhanov R, Rangnekar VM, Wang G, et al (2008) Critical role of prostate apoptosis response-4 in determining the sensitivity of pancreatic cancer cells to small-molecule inhibitor-induced apoptosis. Mol Cancer Ther 7: 2884–2893. 18790769 10.1158/1535-7163.MCT-08-0438 PMC3766350

[27] El Guendy N, Rangnekar VM (2003) Apoptosis by Par-4 in cancer and neurodegenerative diseases. Exp Cell Res 283: 51–66. 12565819 10.1016/s0014-4827(02)00016-2

[28] Boehrer S, Chow KU, Beske F, Kukoc-Zivojnov N, Puccetti E, et al (2002) In lymphatic cells par-4 sensitizes to apoptosis by down-regulating bcl-2 and promoting disruption of mitochondrial membrane potential and caspase activation. Cancer Res 62: 1768–1775. 11912153

[29] Qiu G, Ahmed M, Sells SF, Mohiuddin M, Weinstein MH, et al (1999) Mutually exclusive expression patterns of Bcl-2 and Par-4 in human prostate tumors consistent with down-regulation of Bcl-2 by Par-4. Oncogene 18: 623–631. 9989812 10.1038/sj.onc.1202344

[30] Batista LF, Roos WP, Christmann M, Menck CF, Kaina B (2007) Differential sensitivity of malignant glioma cells to methylating and chloroethylating anticancer drugs: p53 determines the switch by regulating xpc, ddb2, and DNA double-strand breaks. Cancer Res 67: 11886–11895. 18089819 10.1158/0008-5472.CAN-07-2964

[31] Benzina S, Altmeyer A, Malek F, Dufour P, Denis JM, et al (2008) High-LET radiation combined with oxaliplatin induce autophagy in U-87 glioblastoma cells. Cancer Lett 264: 63–70. 18329790 10.1016/j.canlet.2008.01.023

[32] Meng QH, Zhou LX, Luo JL, Cao JP, Tong J, et al (2005) Effect of 7-hydroxystaurosporine on glioblastoma cell invasion and migration. Acta Pharmacol Sin 26: 492–499. 15780200 10.1111/j.1745-7254.2005.00087.x

[33] Patel S, DiBiase S, Meisenberg B, Flannery T, Patel A, et al (2012) Phase I clinical trial assessing temozolomide and tamoxifen with concomitant radiotherapy for treatment of high-grade glioma. Int J Radiat Oncol Biol Phys 82: 739–742. 21353747 10.1016/j.ijrobp.2010.12.053

[34] Hui AM, Zhang W, Chen W, Xi D, Purow B, et al (2004) Agents with selective estrogen receptor (ER) modulator activity induce apoptosis in vitro and in vivo in ER-negative glioma cells. Cancer Res 64: 9115–9123. 15604281 10.1158/0008-5472.CAN-04-2740

[35] Vetterkind S, Boosen M, Scheidtmann KH, Preuss U (2005) Ectopic expression of Par-4 leads to induction of apoptosis in CNS tumor cell lines. Int J Oncol 26: 159–167. 15586236

[36] Lee JW, Lee KF, Hsu HY, Hsu LP, Shih WL, et al (2007) Protein expression and intracellular localization of prostate apoptosis response-4 (Par-4) are associated with apoptosis induction in nasopharyngeal carcinoma cell lines. Cancer Lett 257: 252–262. 17881119 10.1016/j.canlet.2007.08.004

[37] Chakraborty M, Qiu SG, Vasudevan KM, Rangnekar VM (2001) Par-4 drives trafficking and activation of Fas and Fasl to induce prostate cancer cell apoptosis and tumor regression. Cancer Res 61: 7255–7263. 11585763

[38] Nalca A, Qiu SG, El Guendy N, Krishnan S, Rangnekar VM (1999) Oncogenic Ras sensitizes cells to apoptosis by Par-4. J Biol Chem 274: 29976–29983. 10514481 10.1074/jbc.274.42.29976

[39] Barkett M, Gilmore TD (1999) Control of apoptosis by Rel/NF-kappaB transcription factors. Oncogene 18: 6910–6924. 10602466 10.1038/sj.onc.1203238

[40] Boehrer S, Chow KU, Ruthardt M, Hoelzer D, Mitrou PS, et al (2002) Expression and function of prostate-apoptosis-response-gene-4 in lymphatic cells. Leuk Lymphoma 43: 1737–1741. 12685825 10.1080/1042819021000006510

[41] Cheema SK, Mishra SK, Rangnekar VM, Tari AM, Kumar R, et al (2003) Par-4 transcriptionally regulates Bcl-2 through a WT1-binding site on the bcl-2 promoter. J Biol Chem 278: 19995–20005. 12644474 10.1074/jbc.M205865200

[42] Kallio A, Zheng A, Dahllund J, Heiskanen KM, Harkonen P (2005) Role of mitochondria in tamoxifen-induced rapid death of MCF-7 breast cancer cells. Apoptosis 10: 1395–1410. 16215679 10.1007/s10495-005-2137-z

[43] Chaudhry P, Singh M, Parent S, Asselin E (2012) Prostate apoptosis response 4 (Par-4), a novel substrate of caspase-3 during apoptosis activation. Mol Cell Biol 32: 826–839. 22184067 10.1128/MCB.06321-11 PMC3272980

[44] Thayyullathil F, Pallichankandy S, Rahman A, Kizhakkayil J, Chathoth S, et al (2013) Caspase-3 mediated release of SAC domain containing fragment from Par-4 is necessary for the sphingosine-induced apoptosis in Jurkat cells. J Mol Signal 8: 2. 23442976 10.1186/1750-2187-8-2 PMC3599610

[45] Matsuoka H, Tsubaki M, Yamazoe Y, Ogaki M, Satou T, et al (2009) Tamoxifen inhibits tumor cell invasion and metastasis in mouse melanoma through suppression of PKC/MEK/ERK and PKC/PI3K/Akt pathways. Exp Cell Res 315: 2022–2032. 19393235 10.1016/j.yexcr.2009.04.009

[46] Block M, Grundker C, Fister S, Kubin J, Wilkens L, et al (2012) Inhibition of the AKT/mTOR and erbB pathways by gefitinib, perifosine and analogs of gonadotropin-releasing hormone I and II to overcome tamoxifen resistance in breast cancer cells. Int J Oncol 41: 1845–1854. 22922893 10.3892/ijo.2012.1591

[47] Tan J, You Y, Xu T, Yu P, Wu D, et al (2014) Par-4 downregulation confers cisplatin resistance in pancreatic cancer cells via PI3K/Akt pathway-dependent EMT. Toxicol Lett 224: 7–15. 24144893 10.1016/j.toxlet.2013.10.008

[48] Joshi J, Fernandez-Marcos PJ, Galvez A, Amanchy R, Linares JF, et al (2008) Par-4 inhibits Akt and suppresses Ras-induced lung tumorigenesis. EMBO J 27: 2181–2193. 18650932 10.1038/emboj.2008.149 PMC2519103

[49] Xiao H, Goldthwait DA, Mapstone T (1994) The identification of four protein kinase C isoforms in human glioblastoma cell lines: PKC alpha, gamma, epsilon, and zeta. J Neurosurg 81: 734–740. 7931620 10.3171/jns.1994.81.5.0734

[50] Diaz-Meco MT, Municio MM, Frutos S, Sanchez P, Lozano J, et al (1996) The product of par-4, a gene induced during apoptosis, interacts selectively with the atypical isoforms of protein kinase C. Cell. 86: 777–786. 10.1016/s0092-8674(00)80152-x 8797824

[51] Zheng A, Kallio A, Harkonen P (2007) Tamoxifen-induced rapid death of MCF-7 breast cancer cells is mediated via extracellularly signal-regulated kinase signaling and can be abrogated by estrogen. Endocrinology 148: 2764–2777. 17363451 10.1210/en.2006-1269

[52] Matsuoka H, Tsubaki M, Yamazoe Y, Ogaki M, Satou T, et al (2009) Tamoxifen inhibits tumor cell invasion and metastasis in mouse melanoma through suppression of PKC/MEK/ERK and PKC/PI3K/Akt pathways. Exp Cell Res 315: 2022–2032. 19393235 10.1016/j.yexcr.2009.04.009

[53] Burikhanov R, Shrestha-Bhattarai T, Qiu S, Shukla N, Hebbar N, et al (2013) Novel mechanism of apoptosis resistance in cancer mediated by extracellular PAR-4. Cancer Res 73: 1011–1019. 23204231 10.1158/0008-5472.CAN-12-3212 PMC3549021

[54] Rah B, Amin H, Yousuf K, Khan S, Jamwal G, et al (2012) A novel MMP-2 inhibitor 3-azidowithaferin A (3-azidoWA) abrogates cancer cell invasion and angiogenesis by modulating extracellular Par-4. PLoS One 7: e44039. 22962598 10.1371/journal.pone.0044039 PMC3433490

[55] Lee AS (2007) GRP78 induction in cancer: therapeutic and prognostic implications. Cancer Res 67: 3496–3499. 17440054 10.1158/0008-5472.CAN-07-0325

[56] Phillips HS, Kharbanda S, Chen R, Forrest WF, Soriano RH, et al (2006) Molecular subclasses of high-grade glioma predict prognosis, delineate a pattern of disease progression, and resemble stages in neurogenesis. Cancer Cell 9: 157–173. 16530701 10.1016/j.ccr.2006.02.019

[57] Sai K, Wang S, Balasubramaniyan V, Conrad C, Lang FF, et al (2012) Induction of cell-cycle arrest and apoptosis in glioblastoma stem-like cells by WP1193, a novel small molecule inhibitor of the JAK2/STAT3 pathway. J Neurooncol 107: 487–501. 22249692 10.1007/s11060-011-0786-z

[58] Campos B, Wan F, Farhadi M, Ernst A, Zeppernick F, et al (2010) Differentiation therapy exerts antitumor effects on stem-like glioma cells. Clin Cancer Res 16: 2715–2728. 20442299 10.1158/1078-0432.CCR-09-1800

[59] Juan Sebastian Yakisich (2012) Challenges and limitations of targeting cancer stem cells and/or the tumor microenvironment. Drugs and Therapy Studies 2.

