# Correction: Expression and Regulation of Prostate Apoptosis Response-4 (Par-4) in Human Glioma Stem Cells in Drug-Induced Apoptosis

**DOI:** 10.1371/journal.pone.0095817

**Published:** 2014-04-21

**Authors:** The PLOS ONE Staff

There is an error in the first sentence of the first paragraph of the “Cell Culture” sub-section of the Materials and Methods. The correct sentence is: Human Neural Glial cell-line (HNGC-2) was generated from human astrocytoma tumor in National Centre for Cell Science (NCCS), Pune.
