# Supplementary figures and images for: Expression and Regulation of Prostate Apoptosis Response-4 (Par-4) in Human Glioma Stem Cells in Drug-Induced Apoptosis
Source: PLoS One. 2014 Feb 11;9(2):e88505. doi: 10.1371/journal.pone.0088505 (PMC3921173; doi:10.1371/journal.pone.0088505)

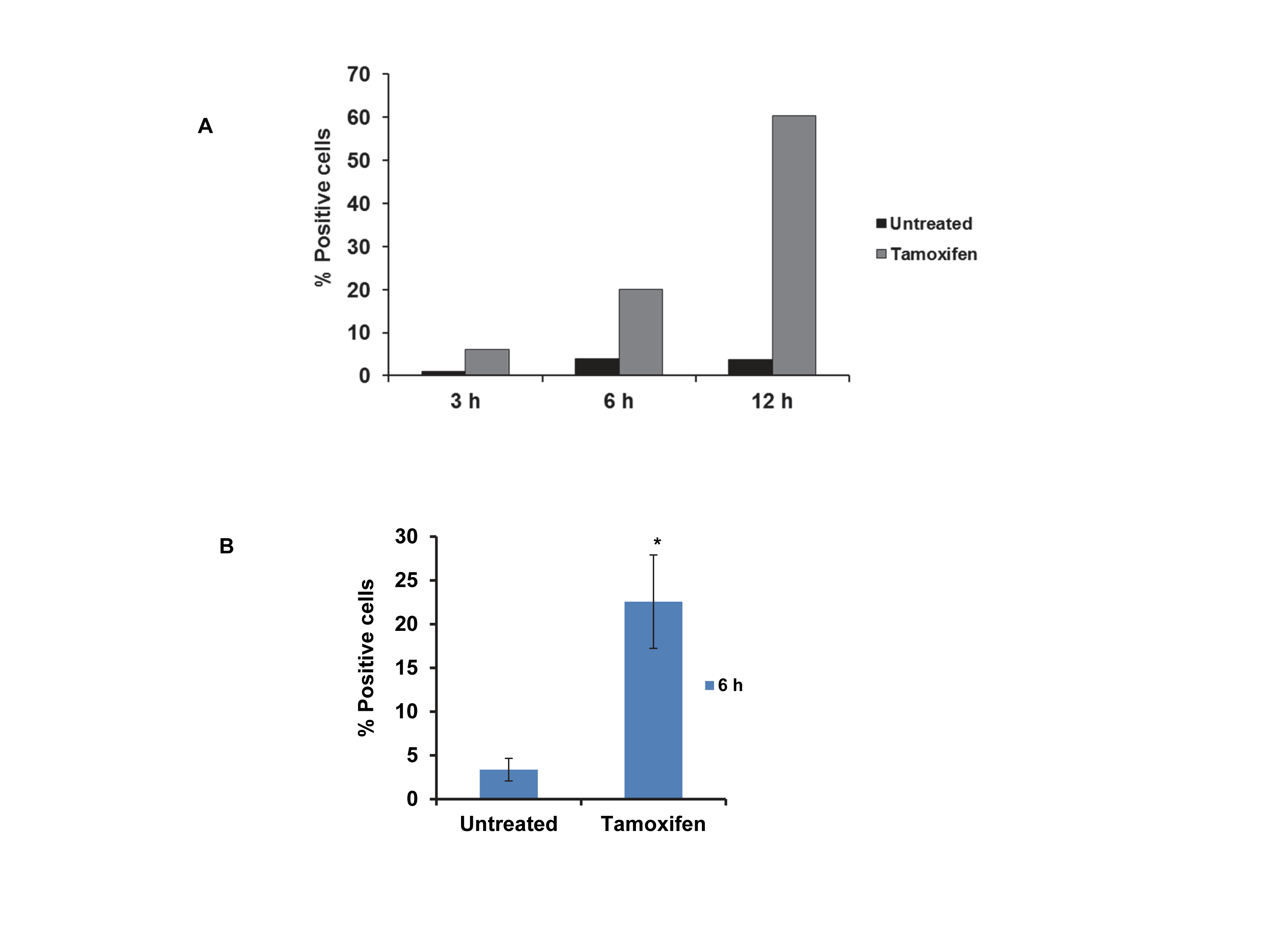

Supplement: Figure S1 — Tamoxifen induced cell death through mitochondrial membrane rupture. (A) HNGC-2 cells were treated by TAM for 3 h, 6 h and 12 h, stained with Mitocapture Reagent (Calbiochem) and analyzed for mitochondrial membrane potential by flowcytometry using FITC channel for green monomers, Ex/Em = 488/530±30 nm (for details refer materials and methods). Y-axis represents percent positive cells for green fluorescence with respect to untreated controls. (B) Mitochondrial membrane potential of HNGC-2 cells, untreated and TAM treated at 6 h were analysed by flow cytometry same as above. Bars indicate mean ± SD (n = 3). * p< 0.018 (TIF) [file pone.0088505.s001.tif]

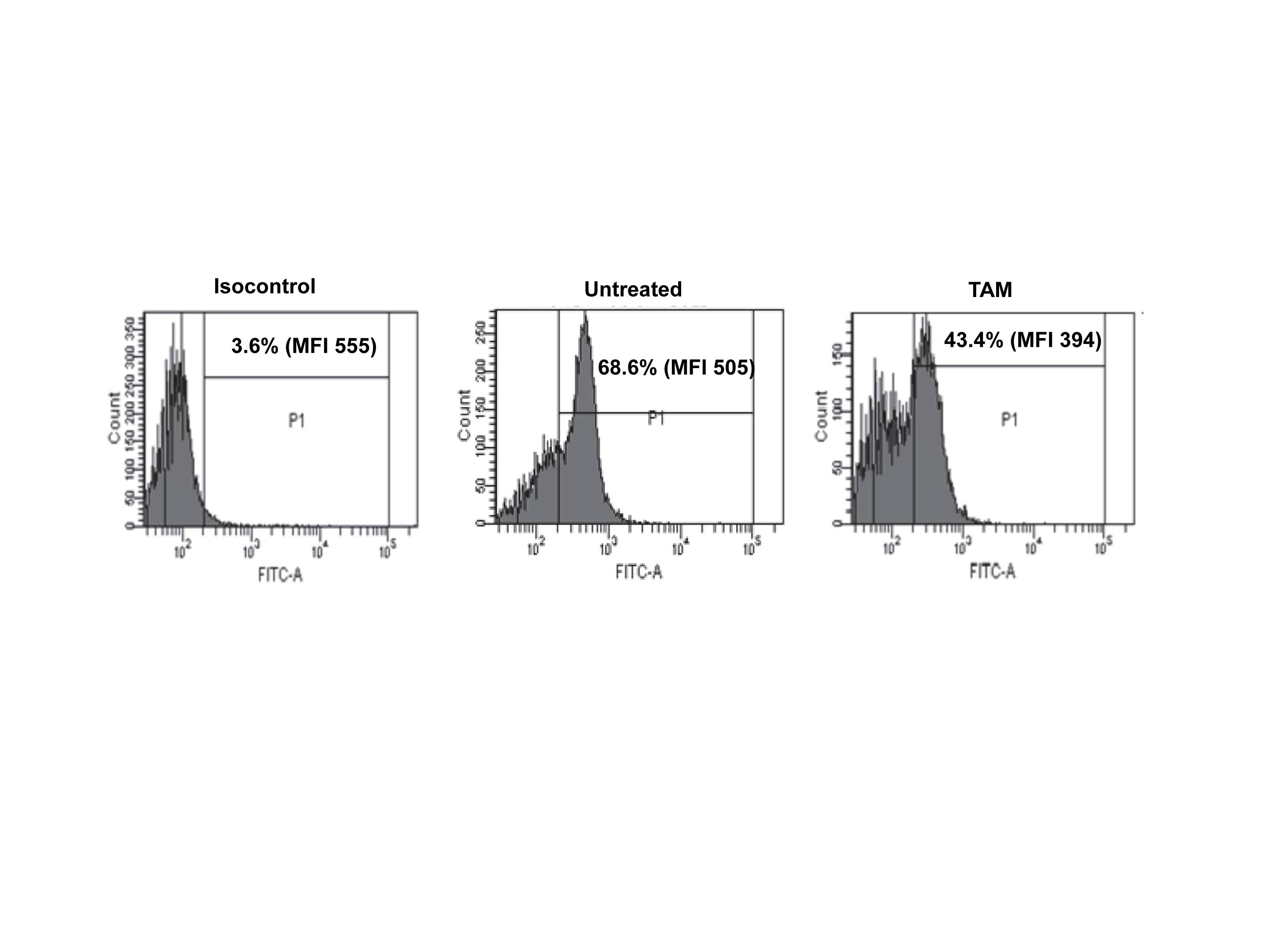

Supplement: Figure S2 — Tamoxifen downregulates Bcl-2 expression in HNGC-2 cells. HNGC-2 cells were treated with TAM for 24 h and expression of Bcl-2 was assessed by Flow cytometry using FITC channel (for details refer materials and methods). Y-axis represents percent positive cells and mean fluorescence intensity (MFI) for green fluorescence with respect to isocontrol. (TIF) [file pone.0088505.s002.tif]

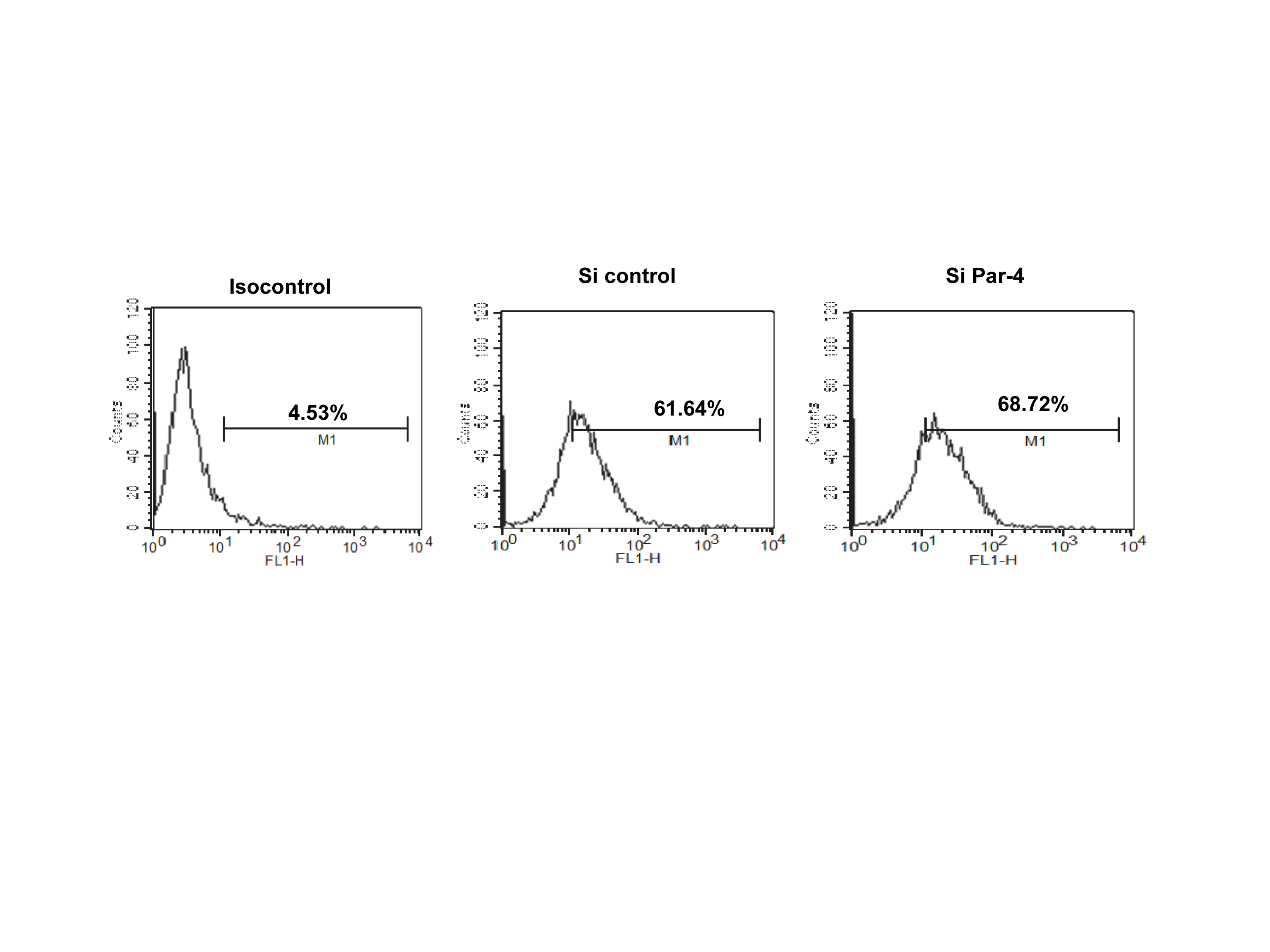

Supplement: Figure S3 — Silencing of Par-4 does not affect Bcl-2 expression significantly in HNGC-2 cells. HNGC-2 cells were transfected with control siRNA and Par-4 siRNAand further analyzed for Bcl-2 levels by flow cytometry (BD Caliber) using FL-1 channel for green fluorescence. Markers in the plots represent percent positive cells with respect to isocontrol. (TIF) [file pone.0088505.s003.tif]

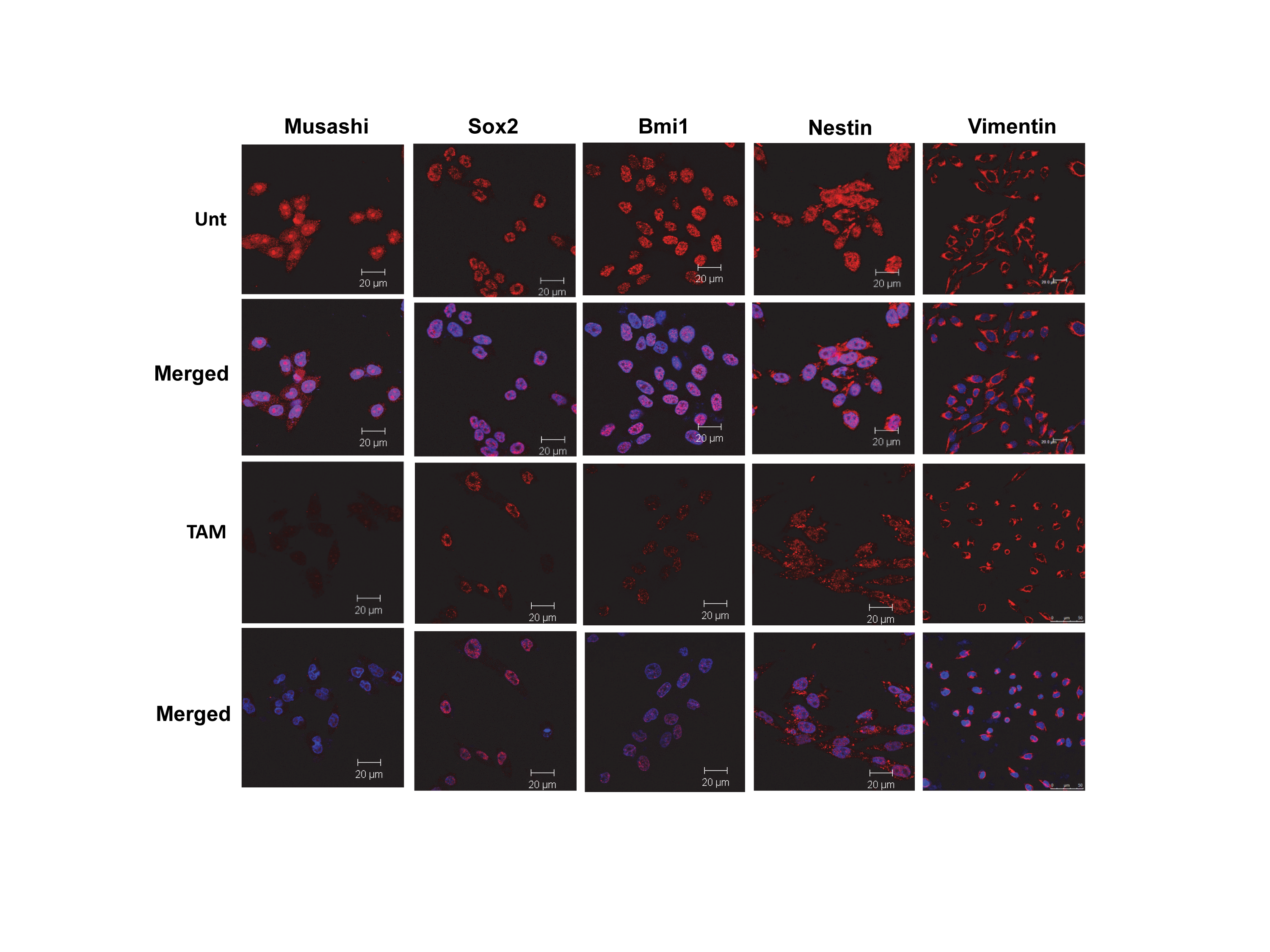

Supplement: Figure S4 — Effect of tamoxifen on stem cell markers in HNGC-2 cells. HNGC-2 cells were treated with TAM and expression of stem cell markers like Bmi1, Nestin, Musashi, Sox2 and Vimentin were visualized using Cy3 secondary antibody (red) using Carl Zeiss/Leica, confocal Microscope (Scale bar - 20µm). (TIF) [file pone.0088505.s004.tif]

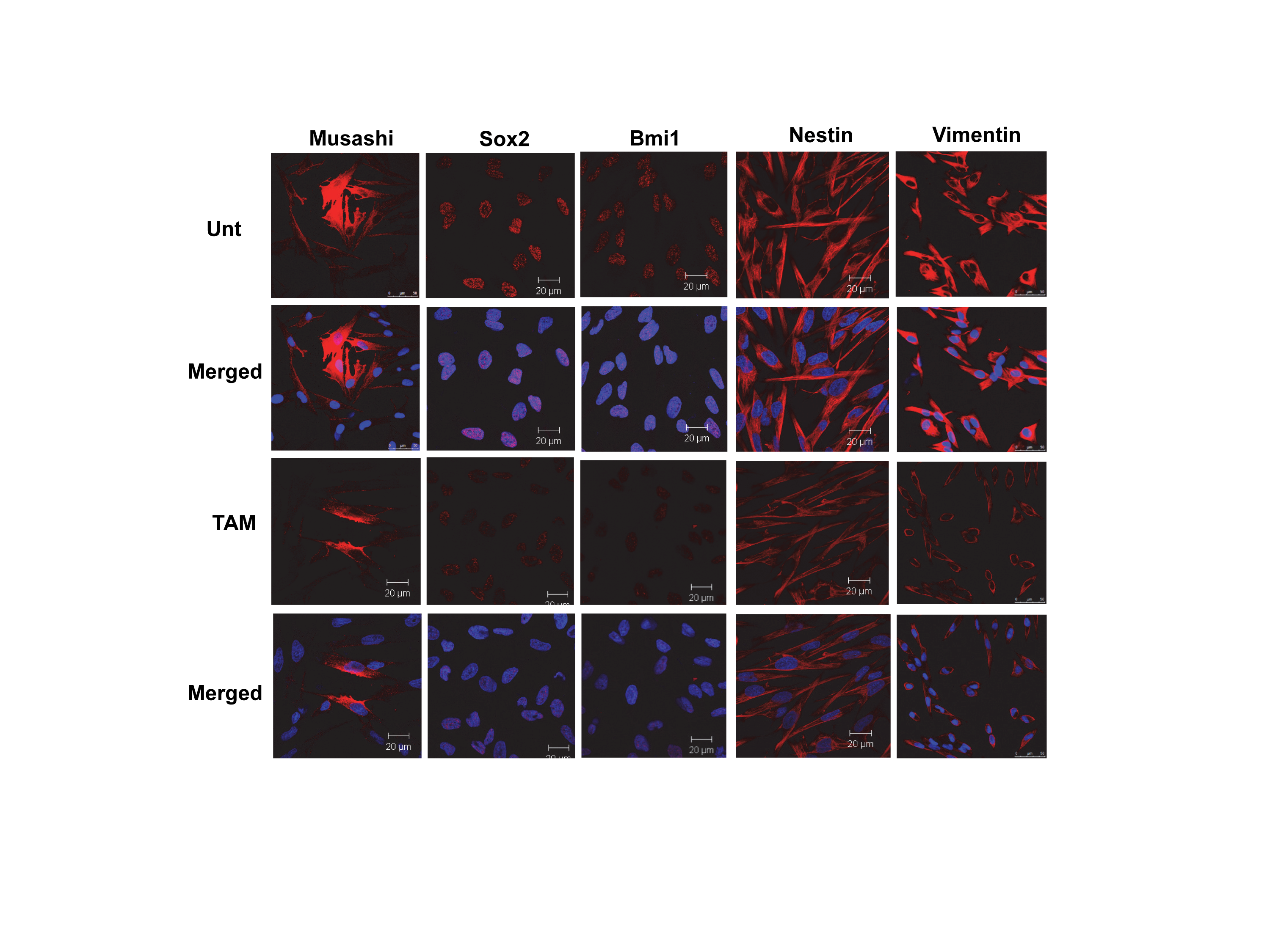

Supplement: Figure S5 — Effect of tamoxifen on stem cell markers in Primary GBM cells (G1). G1 cells were treated with TAM and expression of stem cell markers like Bmi1, Nestin, Musashi, Sox2 and Vimentin were visualized using Cy3 secondary antibody (red) using Carl Zeiss/Leica, confocal Microscope (Scale bar - 20µm). (TIF) [file pone.0088505.s005.tif]

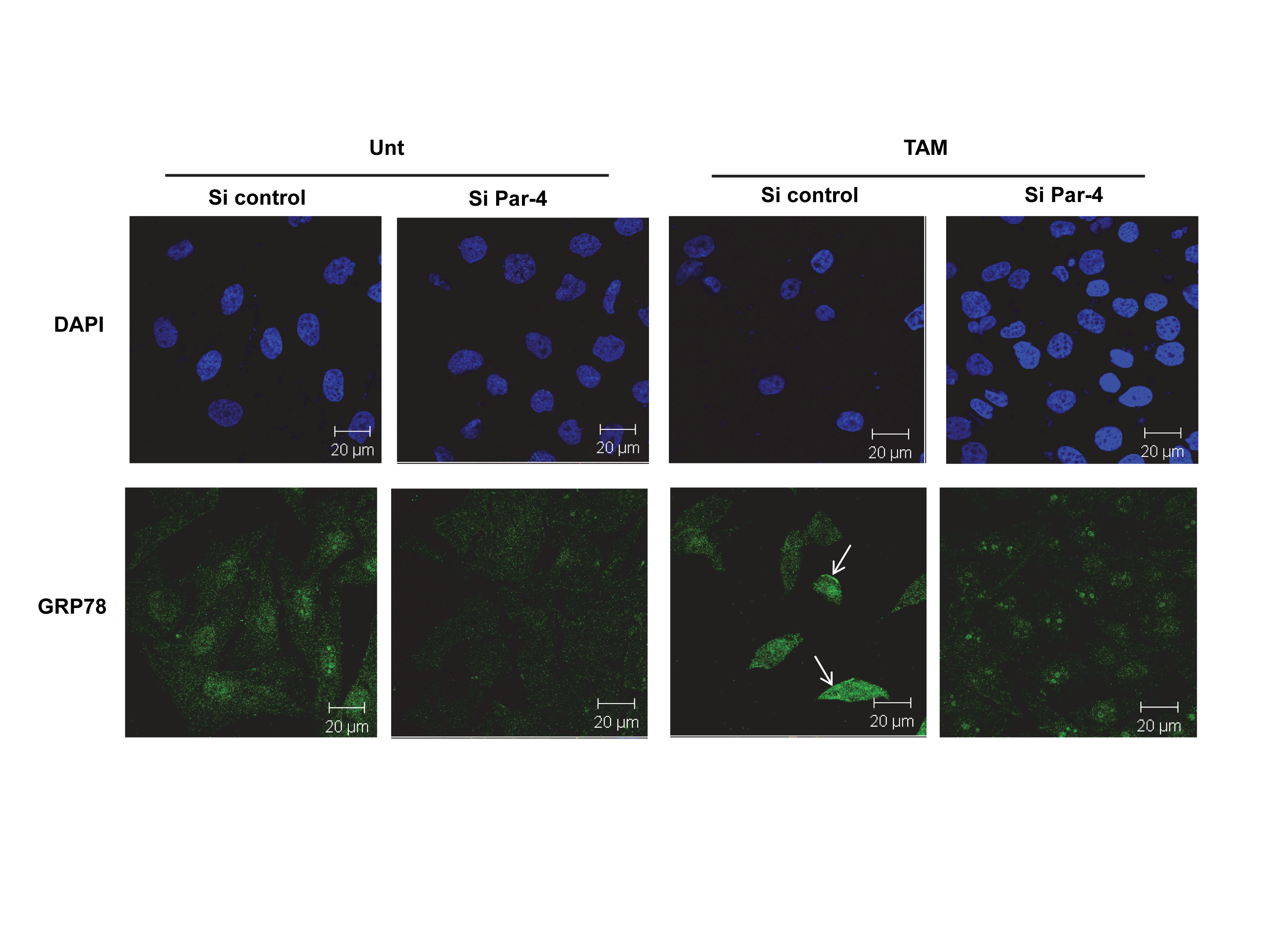

Supplement: Figure S6 — Translocation of GRP78 to the membrane depends on Par-4 levels in HNGC-2 cells. Par-4 siRNA transfected cells were treated with tamoxifen and visualized for GRP78 (green) expression and localization by immunofluorescence (Scale bar - 20µm). (TIF) [file pone.0088505.s006.tif]
